# Web-based Discussion Forums on Pregnancy Complaints and Maternal Health Literacy in Norway: A Qualitative Study

**DOI:** 10.2196/jmir.5270

**Published:** 2016-05-26

**Authors:** Eva Haukeland Fredriksen, Janet Harris, Karen Marie Moland

**Affiliations:** ^1^ Department of Occupational Therapy, Physiotherapy and Radiography Faculty of Health and Social Sciences Bergen University College Bergen Norway; ^2^ School of Health & Related Research University of Sheffield Sheffield United Kingdom; ^3^ Centre for International Health Faculty of Medicine and Dentistry University of Bergen Bergen Norway; ^4^ Department of Nursing Faculty of Health and Social Sciences Bergen University College Bergen Norway

**Keywords:** qualitative research, Internet, pregnancy, health literacy, web-based discussion forums, pelvic girdle pain

## Abstract

**Background:**

The Internet is one of the fastest growing information sources for pregnant women and seems to be used across social and economic strata. However, we still lack knowledge on how interaction in Web-based discussion forums influence maternal health literacy, in terms of how pregnant women access, appraise, and apply information to promote and maintain good health.

**Objective:**

The aim of this study was to understand how Web-based discussion forums influence maternal health literacy; hence, we explored the role of interactions in Web-based discussion forums among women who experienced health problems during pregnancy. More specifically, we explored why media-literate women experiencing the medically unexplained condition, pelvic girdle pain (PGP), during pregnancy participated in Web-based discussion forums and how they appraised and applied the information and advice that they gained from the Web-based interaction with other women.

**Methods:**

Women were invited to participate in the study via postings on 3 different open websites for pregnant women and mothers. The sample included 11 Norwegian women who participated in open Web-based discussion forums when experiencing PGP in pregnancy. The data were collected using synchronous qualitative email interviews and were analyzed using thematic analysis.

**Results:**

In our study sample, interaction in Web-based discussion forums influenced maternal health literacy in terms of increased health-related knowledge and competencies, increased awareness of health promotion and health protection, and increased system navigation. The women appraised and selectively applied information and advice that resonated with their own experiences. For many, the information provided online by other women in the same situation was valued more highly than advice from health professionals. Women reported that they used their knowledge and competency in encounters with health professionals but hesitated to disclose the origin of their knowledge. Those with a high level of education in medicine-related fields raised a concern about the Internet as a source of horror stories and erroneous information and were actively engaged in trying to minimize potential negative effects, by providing biomedical information.

**Conclusions:**

The popularity of Web-based discussion forums among pregnant women suggests that this group needs additional sources of information and support to complement traditional consultations with the health professionals. The professionals need to recognize that pregnant women access Web-based discussion forums for support and information to increase their ability to take better health decisions for themselves. This is a potential resource that health professionals may find useful in consultations with pregnant women.

## Introduction

The Internet is one of the fastest growing sources of information on pregnancy-related health topics and plays a significant role in health information seeking and decision-making, as well as for social networking and support [1,2]. People go online for different purposes. Some look for information or look for other people's stories. Others present their own stories or communicate with people in similar situations to access support and make sense of information [3-6]. People also provide online health support outside the professionals' domain [6-8], and electronic peer-to-peer support groups provide people with unprecedented opportunities to share information and become experts in their condition [4,9]. Pregnant women access the Internet to obtain information [10-12] and gain more control over decisions affecting pregnancy [10]. A study conducted in 2010 reported that the majority participated in Web-based pregnancy discussion groups more than 10 times during pregnancy [2]. A follow-up study from 2011 found that, for many women, Web-based pregnancy discussion groups assisted their decision-making [10].

In Norway, almost everyone has access to the Internet, and most people have access to more than one information and communication technology platform. According to 2015 statistics, 96% of the population in the 16-79 year age group were Internet users, and 90% of the women in the 25-34 year age group used the Internet to seek for health-related information [13]. Web-based discussions forums are popular health information sources for pregnant women; however, we lack knowledge on how they use such discussion forums to increase their health literacy.

“Health literacy” (HL) is a term introduced in the 1970s, but there is still not one universally accepted definition for this term [14]. In 1988, World Health Organization defined HL as “The cognitive and social skills which determine the motivation and ability of individuals to gain access to, understand, and use the information in ways which promote and maintain good health” [14]. Interest in Internet HL (eHealth literacy) has focused on being able to access relevant information from electronic sources and being able to make sense of it. Norman and Skinner define eHealth literacy as the ability to seek, find, understand, and appraise health information from electronic sources and apply the knowledge gained to address or solve a health problem [15]. Their definition rests on Nutbeam’s model, which describes 3 levels of HL, progressing from basic skills in reading and writing (functional HL), to the ability to derive meaning from different forms of communication and apply new information to changing situations (interactive HL), and to the ability to achieve policy and organizational changes (critical HL) [14,16-19]. Nutbeam and Renkert introduced the concept of “maternal health literacy” [20]. This concept is about interactive HL, describing the “cognitive and social skills which determine the motivation and ability of women to gain access to, understand, and use information in ways that promote and maintain their health and that of their children” [20]. In this study, we used a combination of the definitions of interactive HL and eHealth literacy to explore how interaction in Web-based discussion forums influences pregnant women's motivation and ability to access, appraise, and apply information to promote and maintain their health.

We have used the medically unexplained condition, pelvic girdle pain (PGP), as a tracer condition because an increasing number of pregnant women, especially in the Scandinavian countries, are diagnosed with PGP [21]. PGP is about pain in the pelvic girdle and lumbar regions [22] and is a common cause of sick leave among Norwegian pregnant women [23]. Women who experience PGP commonly experience diminished endurance capacity for everyday life activities, such as standing, walking, and sitting [22]. We explored why media-literate Norwegian women who experienced PGP during pregnancy participated in Web-based discussion forums and how they appraised and applied the information and advice that they gained from Web-based interactions with other women.

## Methods

### Synchronous Qualitative Email Interviews

We decided to collect data by using email interviews because this method of data collection enabled women from a wide geographical area to participate in interviews without making any special arrangements. Email interviews also enabled us to recruit women who were juggling work with pregnancy or motherhood and who might view online interviews as less of a burden than face-to-face interviews. We also wanted answers in a here-and-now dialogue between the interviewees and the researcher and decided to use the method of synchronous email interviews. This interview method is described in detail in the Data collection and Analysis section. Next, we developed a thematic semistructured interview guide with 5 main topics: (1) motivation for participation in Internet discussions on PGP, (2) learning outcome, (3) support, (4) encounters with the health professionals, and (5) Internet discussions and health behavior. The interview guide was used as a flexible tool during the interview process.

### Sampling

Invitations to participate in the study were posted on 3 different open websites for pregnant women and mothers. We sought computer-literate women who could read, write, and understand Norwegian, who experienced PGP during pregnancy, and who had participated in Web-based discussion forums during pregnancy. However, we experienced that sampling via the Internet was more challenging than expected. It took us more than 5 months to set up a sample of 11 women who fulfilled the inclusion criteria for the study. Twenty-three women expressed interest, and 11 of them participated in the study. Among the nonparticipants, 7 did not respond to follow-up mails, and 5 were excluded because they did not meet the inclusion criteria.

The first author received permission from the people in charge of the open website www.barnimagen.com to advertise for participants on the discussion forum on PGP, and the invitation was posted in October 2013. During the next 2 months, 10 women made contact and expressed their interest. We emailed invitations with detailed project information and a consent form, but 3 women never responded to the initial or follow-up emails. Seven women responded positively and participated in email interviews during autumn 2013. Next, we decided to post the invitation on an additional Internet site to increase the number of participants. The first author received permission from the midwife in charge of the site www.altformamma.com to advertise for participants. The invitation was posted in December 2013, and it was reposted several times over the next months to generate more informants. During this period, 13 women contacted us. Among those, 5 did not fulfill the inclusion criteria, and 4 did not respond to follow-up emails. Four women were included in the study, with interviews taking place between January and March 2014. To increase the number of participants, we also advertised for participants on the website www.babyverden.com in February 2014. However, there were no responses to our advertisement as it was difficult to see among the large number of threads and discussions on this website. After the final interviews in March, we decided to stop further sampling because although the sample was small, we had quite a rich and nuanced dataset, representing women from different social and professional backgrounds, geographical areas, age groups, and pregnancy status.

### Participants

All the women had participated in Web-based discussions on PGP, and all had experienced PGP during their pregnancies. Most of them were on maternity leave, or were sick-listed due to PGP, and many experienced persisting symptoms of PGP after delivery. We have given nicknames to the participants so that the readers can discriminate between them while reading the Result section.

**Table 1:** Participants.

Participant	Age, years	Profession	Pregnancy status	Nonpregnant women: time since delivery	PGP^a^experience
Anne	28	Secretary	First pregnancy		PGP in pregnancy, sick-listed
Betty	30	Engineer	Second pregnancy		PGP in both pregnancies, sick-listed
Cecilia	29	Nurse	Not pregnant, 2 children	16 weeks	PGP in both pregnancies, persisting symptoms
Dea	24	Bank employee	Third pregnancy		PGP in her last 2 pregnancies
Eva	36	Veterinary	Second pregnancy		PGP in both pregnancies
Frida	39	Assistant nurse	Not pregnant, 2 children	4 years	PGP in both pregnancies, persisting symptoms
Gill	39	Medical doctor	Not pregnant, 1 child	18 months	PGP in pregnancy, persisting symptoms
Hannah	22	Health care assistant	Not pregnant, 1 child	3 months	PGP in pregnancy, persisting symptoms
Inga	38	Farmer	Not pregnant, 1 child	18 months	PGP in pregnancy, persisting symptoms, sick-listed
Jenny	36	Cleaner	Not pregnant, 3 children	6 months	PGP in all pregnancies, persisting symptoms
Karen	22	Kiosk worker	Not pregnant, 1 child	6 months	PGP in pregnancy

^a^PGP: pelvic girdle pain.

### Data Collection and Analysis

All email interviews were carried out in Norwegian by the first author. The interview situation lacked the body language, gestures, smiles, eye contact, and small talk that occur in face-to-face interviews; hence, it demanded a different approach to establish trust and dialogue. The researcher sent follow-up responses with encouraging comments to the participants during the interviews to establish trust and facilitate communication. However, extra care was taken about how the conversation was phrased throughout the interviews, as the researcher was aware that the research participants might use the email correspondence for their own purposes.

One topic was addressed at a time. After receiving the answers on 1 topic, follow-up questions were sent to ask for more information or to prevent misunderstandings, before turning to the next topic. Sometimes, short summaries were written, asking the woman to confirm her interpretation of their answers. This worked well. Many women contributed with detailed answers, and the follow-up questions generated additional information on each topic. Most interviews took 1 to 2 hours, and there were no dropouts. One interview was interrupted by grocery shopping, and another was delayed owing to sick children. These incidents were solved by taking pauses or by completing the interview the next day.

The first author transferred the email correspondence to text files after the interviews, and then she deleted the mails. The Norwegian authors read the interview documents in full text, before the first author performed the initial coding of the data, using Nvivo10. In the initial part of the analysis process, the first author used the concepts of interactive HL [15-17,20] to guide the analysis and identify themes, looking for topics related to motivation and ability to seek, find, understand, appraise, and apply Web-based discussion forum information to changing situations, in ways that promote and maintain health during pregnancy. Thereafter, the team worked together, performed a thematic analysis, based on the initial organization of the data, and reorganized the data under new themes and key themes.

### Ethics Approval

Written informed consent was obtained from all the participants. Ethical approval was given by the Regional Committee for Research Ethics (reference number 2012/2225/REK Vest). We have carefully sheltered the anonymity of all the participants in the study, and the data are stored in a password-based research server, accessible only to the research team.

## Results

### Key Themes

After the analysis process described earlier, we organized the findings into 3 key themes: (1) seeking experience-based health information online; (2) understanding and appraising experience-based online health information; and (3) taking control over one' health. The quotes that illustrate the findings were translated to English by the Norwegian authors.

### Seeking Experience-Based Health Information Online

This theme describes why the participants looked for experience-based health information online and how they judged the relevance of this information. The theme resonates with central dimensions of interactional HL, such as seeking, finding, and appraising relevance of information from different forms of communication.

Most women went online to look for people in the same situation as themselves. Some were primarily looking for information, whereas others were looking for emotional support, detailed advice about special topics, or alternative courses of action for themselves. The expectations and motives to participate in the Internet discussions differed across the sample according to previous experience with PGP. Those without previous experience with PGP, such as Hannah and Karen, sought information and emotional support. Hannah attended the discussion forum because she was worried and needed advice: “*It is good to read that other women are in the same situation (…). If I wonder about something, I always get answers.*” Karen looked for information and practical ideas from women in the same situation:

“I got advice from my mom and my mother in law, but it is a long time since they had babies and they may have forgotten a lot. I feel much better discussing my problems with women who are in the same situation.”

However, pregnant women who had former experience of PGP also sought support and advice from others. Betty wrote: “*I want to meet women who have similar experiences so that I can learn how they managed their pain and what kind of adjustments they made. For instance, what was their threshold for requesting sick leave?*”

Dea participated to diagnose her own symptoms, and to “*receive advice from others who are, or have been in, a similar situation. Above all, I participate because it is best to get advice from people in the same situation as myself.*” Others, as Inga, were primarily looking for emotional support: “*I participate to feel less lonely and to talk with others in the same situation as myself.*”

Others wanted to give support and share knowledge with others, to help them solve their health problems. Many women who gave medical information and advice to others had no professional background from medicine-related fields. The engineer, Betty, for example, said: “*The reason why I have participated in the Internet discussions is that I want to help other women so that they do not have to go through the same distressing experience as I did.*”

Women such as Eva, with high education in medicine-related fields, justified their participation differently: “*I answer if I can contribute with good information. I know much about the medical background of PGP and I have also experienced PGP myself.*”

The women found what they were looking for, appraising the information and support as relevant because it was experience-based and updated and originated from women in the same situation as themselves.

### Understanding and Appraising Experience-Based Online Health Information

This theme describes how interaction with others influenced the participants' knowledge on how to deal with their health problems and how they appraised the validity of the health information that they derived from Web-based interactions. Thus, the theme resonates with other dimensions of interactional HL, such as understanding and appraising validity of information to address or solve a health problem.

The discussion forums seemed to contribute to increased knowledge and higher capability to act on health problems. The women informed each other about symptoms, incidence, and prognosis. Dea said that the Web-based discussions were vital for her knowledge about PGP: “*I think that as much as 80% of my first knowledge about PGP was via other women online.*”

However, this knowledge also made them more aware of potential health risks and of risk reduction. Women reported that the Internet forums had informed them that pushing their physical activity limits during pregnancy might increase the risk of developing persisting health problems after delivery. Such stories fostered an awareness of risks. Women posted directions on where to seek help and treatment and how to request health care or sick leave. However, the advice-seeking women reported that they compared other women's experiences to their own experiences and symptoms before they decided whether to implement the advice.

For many, the advice from others in the same situation fostered an increased understanding of how to maintain health and well-being. Betty had learnt “*what to do to avoid massive health problems in this pregnancy,*” and Karen thought that her new knowledge might help her in her next pregnancy, “*Then I know what to do to avoid massive health problems such as those that I have experienced during this pregnancy. I will be more prepared, so to say.*” Moreover, they had learnt the importance of requesting professional help to prevent worsening of the symptoms. Betty commented that she had learnt what others normally received help for and what she might request from the doctor. Anne described how the Internet forum had given her information on where to find help to cope with the situation and how to describe her symptoms to get adequate health care: “*I have learnt what to tell to receive the help that I need.*”

Women also shared information about social rights, such as access to treatment, physical remedies, job adjustments, or sick leave. This information was highly valued. Some commented that the doctors often lacked an overview of social rights, and that women need to know their rights to receive adequate help. Inga, among others, blamed herself for the persisting health problems: “*If I had known earlier what I know now, I would have demanded more support from the health professionals. Then I could have coped better with the problems.*”

A few women, such as Eva and Gill, who both had high education in medicine-related fields, were concerned about the validity of experience-based knowledge. Eva said that she contributed to the discussions with what she termed “correct information”: “*Sometimes people write horror-stories, and contribute with incorrect information. Then I think it is important to add correct information to the discussions.*” Gill commented that she did not find the information on the discussion forums trustworthy unless it was linked to reliable sources. Other women were less concerned about validity:

“Some answers might have poor quality. But I think that there is valuable advice online, especially advice from others in the same situation who have new experiences. I think that this advice is often better than advice from health professionals, who lack this bodily experience themselves.” (Dea)

Most women developed increased understanding of how to maintain health and well-being in pregnancy, and they appraised the validity of experience-based advice from peers.

### Taking Control Over Own Health

This theme describes how the participants took control over their own health by applying information to promote and maintain their health and illustrates another central dimension of maternal HL.

Many participants described how they navigated in the health system, how they negotiated with the health professionals to get their needs met, and how they applied the advice from the Internet to cope with their everyday life. Some reported how they had contacted doctors and requested physiotherapy, chiropractic services, or acupuncture treatment. Anne, for example, wrote: “*Participation in these discussions has convinced me that I have PGP. That is the reason why I took the next step, and contacted a doctor.*” Jenny negotiated with her doctor to receive treatment for PGP while pregnant, arguing that she was eligible to the same social rights as other women in her situation. She also said that she had learnt what to request if her health problems worsened. Other women shared this experience of being empowered. They commented that support and advice from other women had made them more determined to request help, adjustment, and treatment. Frida said that when she had requested physiotherapy treatment, she did not give in until the doctor responded to her requests. Eva thought that because of her background, she had different experiences: “*My impression is that they listen differently and better to me because I have medical education, which enables me to argue for my needs.*” Nevertheless, she also valued information on treatment options and had followed advice to seek physiotherapy treatment. The medical doctor, Gill, found it interesting to participate in the Internet discussions, but she was clear that this did not influence her health behavior.

However, some women did not argue for their needs at all. They said that they hid their knowledge and its origins in the encounter with the health care providers because they knew that the professionals did not value experience-based knowledge from Internet forums. Dea told that she never shared her thoughts and knowledge with the health care providers because she had experienced that the professionals did not listen to her: “*I am only saying that I know nothing.*” Karen also kept her knowledge to herself: “*I have never received positive feedback from my doctor when I have told what I have read on the Internet (…). He said that most online information was wrong.*” Dea and Karen took control over their health by using a strategy of covert resistance vis-a-vis the health professionals, using advice from others that resonated with their own experiences. Hannah also reported how Internet discussions had enabled her to get better health control. She reported that she had learnt that she should relax and let other people do things for her:

“I have good health control now, as a result of good advice from other women on the Internet. (…) I have used good exercises and pelvic belt, relaxed when I had pain, and accepted that it is ok to stay in bed if needed.”

The data indicate selective application of knowledge, as the women appraised and applied information and advice that resonated with their own experiences.

## Discussion

### Principal Findings

In our study sample, interaction in Web-based discussion forums influenced maternal HL, in terms of increased health-related knowledge and competencies, increased awareness of health promotion and health protection, and better capability to navigate health systems. Women critically appraised and selectively applied information and advice that resonated with their own experiences. For many, the information provided online from other women in the same situation was valued more highly than advice from health professionals. Women reported that they used their knowledge and competency in the encounter with health professionals but hesitated to disclose the origin of their knowledge. Those with a high level of education in medicine-related fields raised a concern about the discussion forums as a source of horror stories and erroneous information and were actively engaged in trying to minimize potential negative effects, by providing biomedical information. Women's concerns about the quality of online pregnancy-related health information have been reported elsewhere [10]. It has also been reported that personal stories from other people in the same situation are not necessarily reliable and can represent both a negative and a positive source of support [24]. However, in our study, concerns about the quality and trustworthiness of the information were generally overshadowed by the positive evaluation of experience-based knowledge and support from others in the same situation.

Our findings on Web-based discussion forums as a source of information and support for pregnant women resonate with findings of other studies in the field [6,10,24]. A study of the role of the Internet in problematic pregnancies reported that women used the Internet as a complementary source for pregnancy information and also to learn from others in similar situations [24]. Several studies have reported that pregnant women turned to technology to fill their knowledge gap [2,10-12,25] and that more informed patients can change the interaction with the health professionals [4,5]. However, previous research has reported diverging findings on whether pregnant women revealed their Internet-based knowledge and discussed this information with the health professionals [2,10-12]. A Web-based survey from 2010, with participants from 24 countries, found that 70% of the participants referred to Internet information in discussion with at least 1 health care provider and that almost 90% of those reported that the information was welcomed and openly discussed [2]. Other studies, from China and Sweden, reported that the majority had not discussed information from the Internet with their health care providers [11,12]. Diverging findings on communication with the health professionals may refer to different cultural norms in terms of the types of knowledge people bring into the medical encounter. However, when discussing communication with the health professionals, the studies referred previously [2,10-12] did not distinguish between different information sources on the Internet. Hence, we do not know whether the women only discussed information that they had retrieved from official health information sites or whether they also discussed information that they had retrieved from informal Web-based discussion forums. Our finding that women actively hide discussion forums as their sources of information was not discussed in the aforementioned studies.

As far as we know, previous Internet research has not explored the influence of informal discussion forums on maternal HL. Women have traditionally shared private concerns and received informal support and advice from other women during pregnancy. This sharing and caring in an informal private space has partly been replaced by informal interaction on the Internet. Interaction with others who share the same illness experiences may have greater weight than advice from health professionals, who are unlikely to have these experiences [6]. Women also tend to listen to other women in the same situation when their embodied experiences diverge from the experts' biomedical knowledge [26,27]. Many participants in our study valued the Web-based information from other women higher than advice from health professionals when the information resonated with their own experiences. The women found the information and support enabling, in terms of controlling their health condition. It has been reported elsewhere that Web-based support can give people a sense of empowerment [6,10,24], and there is increasing evidence that hearing other people's stories can affect health behavior [6]. In general, support from others may have positive health effects because it may increase motivation for self-care and encourage help-seeking behavior and may thus prevent minor illnesses from developing into more serious conditions [28]. However, peer support may also encourage healthy people to request medical assistance for normal life events and minor health problems and may thus contribute to increased medicalization [28]. More seriously, hearing about other people's experiences may reinforce unhealthy behavior [2,6], influencing others to make inappropriate decisions about the management of their symptoms. However, our findings indicate that pregnant women critically appraised and selectively applied information and advice from the Internet and used the advice that resonated with their own experiences.

### Strengths, Challenges, and Limitations

Using email interviews produced a sample that included women from a wide geographical area and different social backgrounds. Many participants commented that communication via email enabled them to participate from their home without making any special arrangements. Other Internet studies on pregnancy and birth have also reported that the participants valued email interviews [29].

The recruitment process was challenging. We tried to increase the number of participants by advertising for participants on several websites. However, this generated answers from women who did not fulfill the inclusion criteria. It was also challenging to create commitment online. Many withdrew from further interaction when they realized that they were expected to follow formal research procedures, including signing a written consent form. Although participants were self-selected, they had in-depth experience of the phenomenon and came from diverse backgrounds. But, the sample was weighted toward women with persisting health problems after pregnancy. We do not know whether currently pregnant women would have different views or whether different views and experiences vary systematically among women from different social and educational backgrounds.

### Conclusions and Implications

In our study sample, interaction in Web-based discussion forums influenced maternal HL. The popularity of Web-based discussion forums among pregnant women suggests that this group needs additional sources of information and support to complement traditional consultations with the health professionals. Health care providers should acknowledge Web-based discussion forums as a source of health information that may influence pregnant women's health behavior. They also need to recognize that pregnant women access Web-based discussion forums for support and information to increase their ability to take better health decisions for themselves. This is a potential resource that health professionals may find useful in consultations with pregnant women.

## Abbreviations

PGP: pelvic girdle pain
HL: health literacy
